# Minimal Matricectomy Combined with Lateral Longitudinal Excision Versus Total Nail Avulsion for Ingrown Toenail: A Two-Center Retrospective Cohort Study with Propensity Score Analysis

**DOI:** 10.3390/medicina62101986

**Published:** 2026-10-14

**Authors:** Ahmet Topcu, İsmail Ege Subaşı, Furkan Saydın

**Affiliations:** Department of General Surgery, Sancaktepe Şehit Prof. Dr. İlhan Varank Training and Research Hospital, Republic of Türkiye Ministry of Health, University of Health Sciences, Istanbul 34785, Türkiye; drahmettopcu36@gmail.com (A.T.); dregesubas@gmail.com (İ.E.S.)

**Keywords:** ingrown toenail, onychocryptosis, matricectomy, nail avulsion, recurrence, propensity score, Kaplan–Meier, surgical outcomes, cohort study

## Abstract

*Background and Objectives*: Ingrown toenail frequently causes pain and infection and may recur following surgical treatment. This study compared a nail-preserving strategy, minimal matricectomy combined with lateral longitudinal excision, against total nail avulsion for recurrence and clinical outcomes, using propensity-score analysis to address non-randomized treatment allocation. *Materials and Methods*: In this two-center retrospective cohort study, 412 patients treated between January 2020 and December 2024 were classified according to the surgical procedure received: minimal matricectomy with lateral longitudinal excision (*n* = 233) or total nail avulsion (*n* = 179). The primary endpoint was recurrence; secondary outcomes included postoperative pain, healing time, infection, reintervention, return to activity, and patient satisfaction. Inverse probability of treatment weighting (IPTW) was used as a sensitivity analysis, and recurrence-free survival was assessed using the Kaplan–Meier method with the log-rank test. *Results*: Over a mean follow-up of 18 months, recurrence occurred in 1.7% (*n* = 4) of the minimal matricectomy group versus 12.3% (*n* = 22) of the avulsion group (*p* < 0.001; crude OR 8.0, 95% CI 2.7–23.7). After IPTW adjustment, the association remained consistent (adjusted OR 7.4, 95% CI 2.6–21.3; standardized mean differences <0.10 for all covariates). Kaplan–Meier analysis confirmed significantly higher recurrence-free survival with minimal matricectomy (log-rank *p* < 0.001). Reintervention (1.3% vs. 9.5%) and return to normal activity (5 vs. 9 days) also favored minimal matricectomy, with lower pain scores, shorter healing, fewer infections, and higher satisfaction. *Conclusions*: Minimal matricectomy combined with lateral longitudinal excision was associated with lower recurrence and improved clinical outcomes compared with total nail avulsion. This association remained statistically significant after propensity score adjustment for measured baseline covariates. Prospective randomized studies are warranted to confirm these findings.

## 1. Introduction

Ingrown toenail, or onychocryptosis, is a frequently encountered pathology of the nail unit that predominantly affects the great toe. The condition arises when the nail plate exerts pressure on or penetrates the adjacent soft tissue, leading to localized inflammation, pain, and in more advanced stages, infection. Beyond its clinical presentation, it may substantially limit daily activities and functional capacity, particularly in younger and physically active individuals [1,2].

A variety of treatment options have been described for ingrown toenail, differing in the extent of tissue removal, mechanism of action, and expected recurrence profile, yet no universally accepted standard approach has been established. Chemical matricectomy with phenol is among the most extensively studied techniques, in which the germinal matrix is selectively destroyed following partial nail plate avulsion; it is valued for its technical simplicity and low cost, although healing may be prolonged due to caustic tissue necrosis [3,4]. The Winograd technique involves surgical excision of the lateral nail plate together with the corresponding matrix horn and adjacent granulation tissue, offering more precise tissue removal than phenolization but requiring greater surgical skill. Radiofrequency ablation has also been employed to achieve matrix destruction with reportedly reduced postoperative discomfort, and CO_2_ laser ablation of the matrix has similarly been proposed as a minimally invasive alternative, although both remain constrained by cost, equipment availability, and limited comparative data [5]. Surgical wedge resection, involving en bloc removal of the lateral nail fold and matrix, provides a more definitive option for severe or recurrent cases but is associated with greater tissue trauma and longer convalescence. Although total nail avulsion has historically been used in the management of ingrown toenail and remains part of clinical practice in some settings, removal of the nail plate without treatment of the underlying matrix or nail-fold pathology does not directly address the anatomical factors contributing to recurrence [6,7,8]. Reported limitations include postoperative discomfort, delayed healing, cosmetic concerns, and recurrence [6,7,8]. Recurrence rates vary considerably according to the procedure performed, follow-up duration, and patient-related factors [3,7,8].

Nail-preserving and matrix-directed surgical principles have long been incorporated into the management of ingrown toenail and continue to be refined in contemporary practice. Rather than removing the entire nail plate, these approaches aim to modify the pathological interaction between the affected nail edge, matrix, and lateral nail fold while preserving unaffected nail structures [4,5,9]. Procedures such as partial matricectomy and targeted soft-tissue excision are based on this principle and aim to reduce recurrence while maintaining functional and cosmetic integrity [5,9,10].

Minimal matricectomy combined with lateral longitudinal excision represents a nail-preserving, matrix-directed surgical approach based on these established principles. The fundamental principle of this approach is to establish a controlled and durable separation between the nail plate and the surrounding soft tissue, thereby minimizing repetitive mechanical irritation. By addressing the structural relationship that underlies disease progression, this technique may offer advantages in preventing recurrence while preserving nail anatomy. Importantly, the clinical question addressed here is whether the outcomes associated with this nail-preserving surgical strategy remain favorable after accounting for measured differences between treatment groups in routine, non-randomized clinical practice.

Despite growing interest in such tissue-preserving methods, direct comparative evidence with total nail avulsion remains limited, particularly in studies reflecting real-world clinical settings and applying confounding-adjustment methods appropriate to non-randomized data. Furthermore, many previously published studies have methodological limitations, including insufficient adjustment for confounding variables and a lack of comprehensive evaluation of patient-related factors such as comorbidities, footwear habits, and nail care practices [5,10,11,12].

Accordingly, the aim of the present study is to describe the real-world outcomes of minimal matricectomy combined with lateral longitudinal excision as applied in routine clinical practice across two centers, including recurrence, postoperative pain, healing time, infection rate, reintervention, return to activity, and patient satisfaction, using total nail avulsion as the comparator procedure performed in the study cohort. To address the non-randomized nature of treatment allocation, a propensity score-based sensitivity analysis and Kaplan–Meier time-to-recurrence analysis were performed in addition to the primary crude comparison and univariate analyses of factors associated with recurrence.

## 2. Materials and Methods

This two-center retrospective cohort study analyzed existing clinical records of patients treated for ingrown toenail at Tokat State Hospital and Sancaktepe Şehit Prof. İlhan Varank Training and Research Hospital between January 2020 and December 2024. Eligible patients treated during the study period were consecutively identified from institutional clinical records according to the predefined inclusion and exclusion criteria and were followed for a minimum of 8 months. A total of 412 patients were included in the analysis and were classified according to the surgical procedure received: minimal matricectomy with lateral longitudinal excision (*n* = 233) or total nail avulsion (*n* = 179). Baseline characteristics, including age, sex distribution, and comorbidities such as diabetes mellitus and obesity, were recorded for both groups. Disease severity at presentation was retrospectively classified according to the Heifetz staging system (Stage I–III) based on documentation in the patient records, and was compared between groups to assess residual baseline imbalance not captured by the variables above.

Inclusion criteria were: a clinical diagnosis of ingrown toenail (onychocryptosis) confirmed on examination; treatment with either minimal matricectomy combined with lateral longitudinal excision or total nail avulsion at one of the two participating centers during the study period; and a minimum follow-up of 8 months with sufficiently complete records to extract the baseline and outcome variables listed above. Exclusion criteria were a surgical technique other than the two procedures under investigation, concurrent conditions expected to materially affect wound healing, and incomplete or missing follow-up data precluding assessment of the primary endpoint.

Patients were categorized into two groups based on the surgical technique applied: minimal matricectomy combined with lateral longitudinal excision and total nail avulsion. Treatment allocation was non-randomized and determined by the operating surgeon based on clinical presentation, institutional practice patterns, and patient preference. While this reflects real-world clinical decision-making, it introduces a potential risk of selection bias, which was addressed analytically as described in Section 2.3.

### 2.1. Surgical Technique

**Figure 1:**
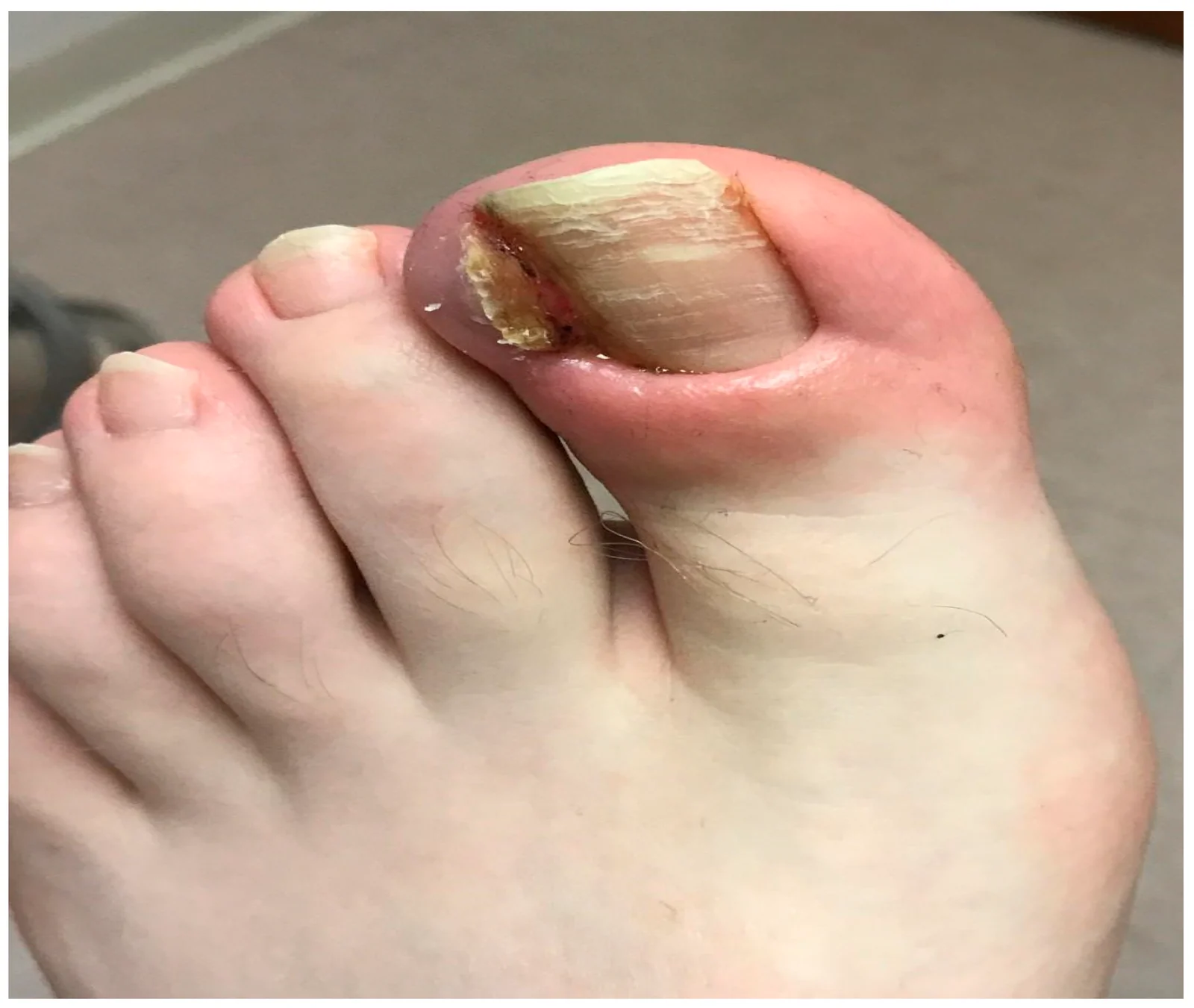
Preoperative clinical appearance of ingrown toenail (onychocryptosis) involving the lateral nail fold, showing inflammation, edema, and granulation tissue formation.

Preoperative appearance of a representative case is shown in Figure 1. Both procedures were performed under digital block anesthesia with 2% lidocaine without epinephrine and a sterile digital tourniquet for hemostasis.

**Minimal matricectomy with lateral longitudinal excision:** A longitudinal strip of the affected lateral nail plate, approximately 2 mm in width, was excised along its full length to the proximal nail fold. The lateral longitudinal soft-tissue excision extends from the lateral nail fold at the level of the distal interphalangeal crease proximally to the eponychial fold, removing the hypertrophic/granulation tissue en bloc. The corresponding germinal matrix segment underlying the excised nail strip is then identified and excised sharply under direct vision (rather than curetted), with hemostasis achieved using bipolar electrocautery. Wound edges are approximated with interrupted 4-0/5-0 nylon sutures, removed at 10–14 days.

**Total nail avulsion:** The entire nail plate is separated from the underlying nail bed and matrix using a periosteal elevator and removed in full, without matrix ablation. In the representative postoperative case, an additional suture was placed for local hemostasis; this was not a routine component of the total nail avulsion technique described above.

**Postoperative care:** A non-adherent absorbent dressing is applied and changed every 48 h until full epithelialization. Antibiotics are not administered routinely and are reserved for clinically evident infection. Patients are permitted immediate full weight-bearing in open or wide-toe footwear, transitioning to regular footwear once comfortable.

**Figure 2:**
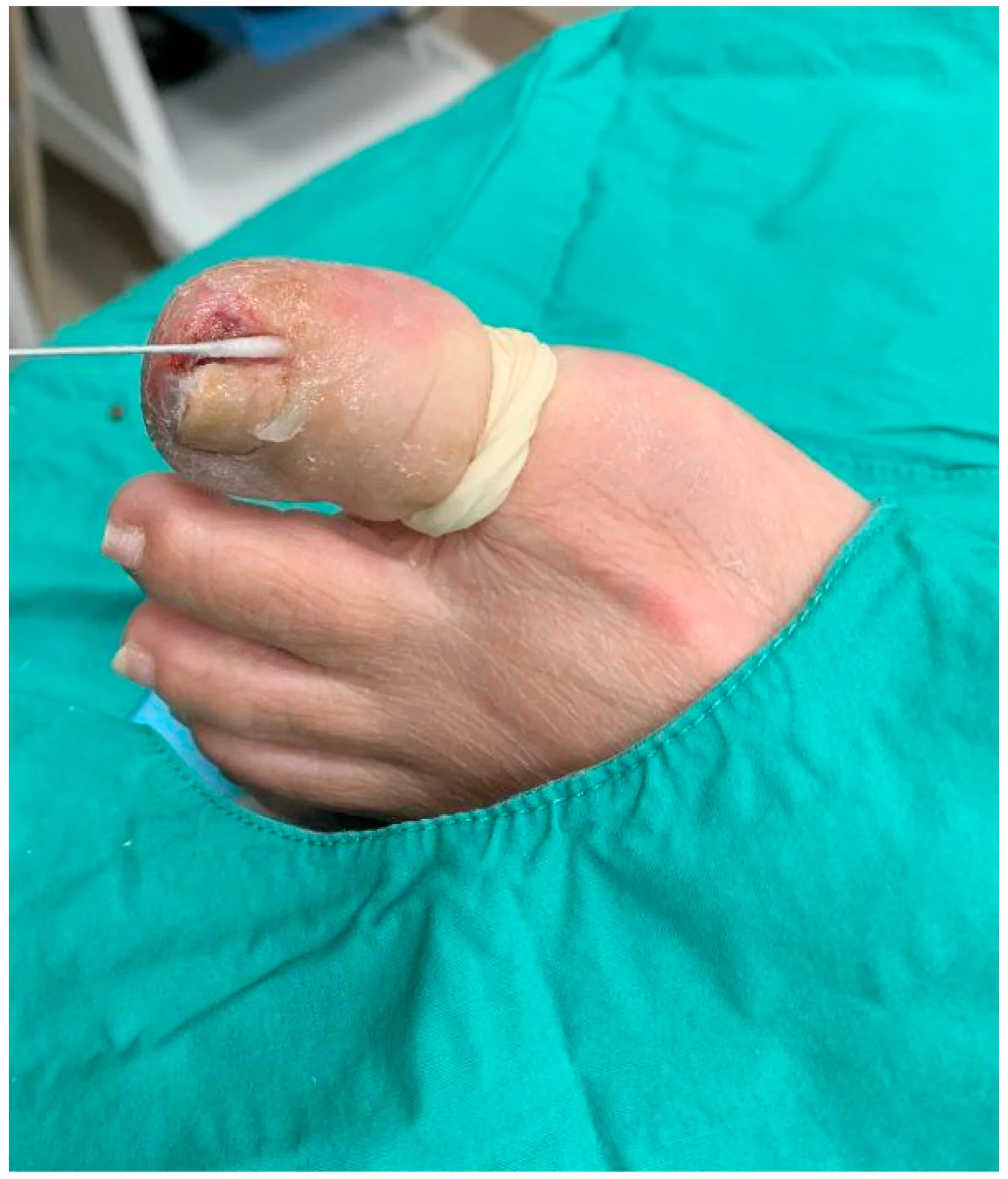
Intraoperative view of minimal matricectomy combined with lateral longitudinal excision. A longitudinal strip of the affected lateral nail plate, approximately 2 mm in width, is excised to the nail root, followed by removal of the corresponding matrix segment. This technique creates a permanent anatomical separation between the nail plate and lateral nail fold. (See Section 2.1 for the full step-by-step description, including anesthesia, hemostasis, suture, and dressing details).

The key steps of the procedure, including lateral longitudinal excision and minimal matricectomy, are demonstrated in Figure 2. Patients were followed for a mean duration of 18 months (range: 8–24 months).

### 2.2. Outcome Definitions

**Primary endpoint—Recurrence:** Recurrence was defined as recurrent penetration of the nail plate into the lateral nail fold associated with pain, inflammation, granulation tissue, or the requirement for repeat intervention, confirmed on clinical examination during follow-up.

Secondary outcomes included postoperative pain assessed by Visual Analog Scale (VAS) on postoperative day 3, healing time defined as complete epithelialization, postoperative infection requiring antibiotic therapy, reintervention rate (defined as any repeat surgical or chemical intervention for the same lesion), time to return to normal activity/work (defined as the point at which the patient resumed unrestricted weight-bearing and usual daily/occupational activity), and patient satisfaction measured using a 5-point Likert scale. These outcomes were selected based on their clinical relevance and frequent use in previous studies evaluating ingrown toenail treatments [10,11,12,13].

### 2.3. Statistical Analysis

Statistical analysis was performed using IBM SPSS Statistics version 26.0 (IBM Corp., Armonk, NY, USA) and R version 4.3.2 (R Foundation for Statistical Computing, Vienna, Austria) (propensity score and survival analyses). Continuous variables were expressed as mean ± standard deviation, and categorical variables as numbers and percentages. Group comparisons were conducted using the chi-square test for categorical variables and Student’s *t*-test or Mann–Whitney U test for continuous variables, as appropriate. Heifetz stage distribution was compared between groups using the chi-square test.

Given the non-randomized allocation of treatment, a propensity score for receiving minimal matricectomy (versus total nail avulsion) was estimated using multivariable logistic regression, including age, sex, diabetes mellitus, obesity/BMI, Heifetz stage, footwear habits, improper nail-trimming practice, treating center, and prior nail intervention as covariates. Stabilized inverse probability of treatment weights (IPTW) were derived from the estimated propensity scores, and covariate balance after weighting was assessed using standardized mean differences (SMD), with SMD < 0.10 considered indicative of adequate balance. The crude (unweighted) comparison was designated the primary analysis, given the modest number of recurrence events, and the IPTW-weighted logistic regression was performed as a pre-specified sensitivity analysis to assess whether the observed association persisted after adjustment for measured confounding. A full multivariable outcome regression was not performed given the low absolute number of recurrence events, which would risk model overfitting and unstable estimates; the IPTW approach was chosen specifically because it addresses confounding at the design stage rather than requiring a heavily parameterized outcome model.

Time to recurrence was additionally analyzed using the Kaplan–Meier method, with groups compared using the log-rank test; patients without recurrence were censored at their last follow-up visit. Results were reported as odds ratios (OR), with 95% confidence intervals (CI), and a *p*-value <0.05 was considered statistically significant.

### 2.4. Ethics

All surgical procedures performed between 2020 and 2024 were undertaken as part of routine clinical care and not as prospectively assigned research interventions. Ethics committee approval was obtained before retrospective extraction and analysis of the clinical data for research purposes, from the Scientific Research Ethics Committee of S.B.U. Sancaktepe Şehit Prof. Dr. İlhan Varank Training and Research Hospital (File No: 10; Date: 14 January 2026). The study was conducted in accordance with the Declaration of Helsinki. As the analysis was retrospective in nature, the requirement for informed consent was waived by the ethics committee.

## 3. Results

A total of 412 patients were analyzed in this study. Baseline demographic and clinical characteristics are presented in Table 1; no statistically significant between-group differences were observed for the reported variables (*p* > 0.05). The mean follow-up duration was 18 months. Heifetz stage distribution did not differ significantly between groups (Stage I: 35.6% [*n* = 83] vs. 33.5% [*n* = 60]; Stage II: 47.6% [*n* = 111] vs. 49.7% [*n* = 89]; Stage III: 16.7% [*n* = 39] vs. 16.8% [*n* = 30]; *p* = 0.87), as shown in Table 2.

The primary outcome analysis demonstrated that the recurrence rate was significantly lower in the minimal matricectomy with lateral longitudinal excision group compared to the total nail avulsion group (1.7% vs. 12.3%, *p* < 0.001). The odds of recurrence were significantly higher in the nail avulsion group (crude OR: 8.0; 95% CI: 2.7–23.7), as shown in Table 3.

In the propensity score-weighted sensitivity analysis, covariate balance was achieved for all measured confounders (SMD < 0.10; Table 4). The IPTW-adjusted odds of recurrence remained significantly higher with total nail avulsion (adjusted OR: 7.4; 95% CI: 2.6–21.3), indicating that the observed association was not substantially explained by the measured baseline covariates.

With respect to secondary clinical outcomes, patients treated with minimal matricectomy exhibited significantly lower postoperative pain scores, shorter healing times, reduced infection rates, and higher levels of patient satisfaction compared to those undergoing total nail avulsion. Reintervention was required in 1.3% (*n* = 3) of the minimal matricectomy group versus 9.5% (*n* = 17) of the total nail avulsion group (*p* < 0.01), and median time to return to normal activity was 5 days versus 9 days, respectively (*p* < 0.001). Detailed comparisons of these parameters are presented in Table 5.

**Figure 3:**
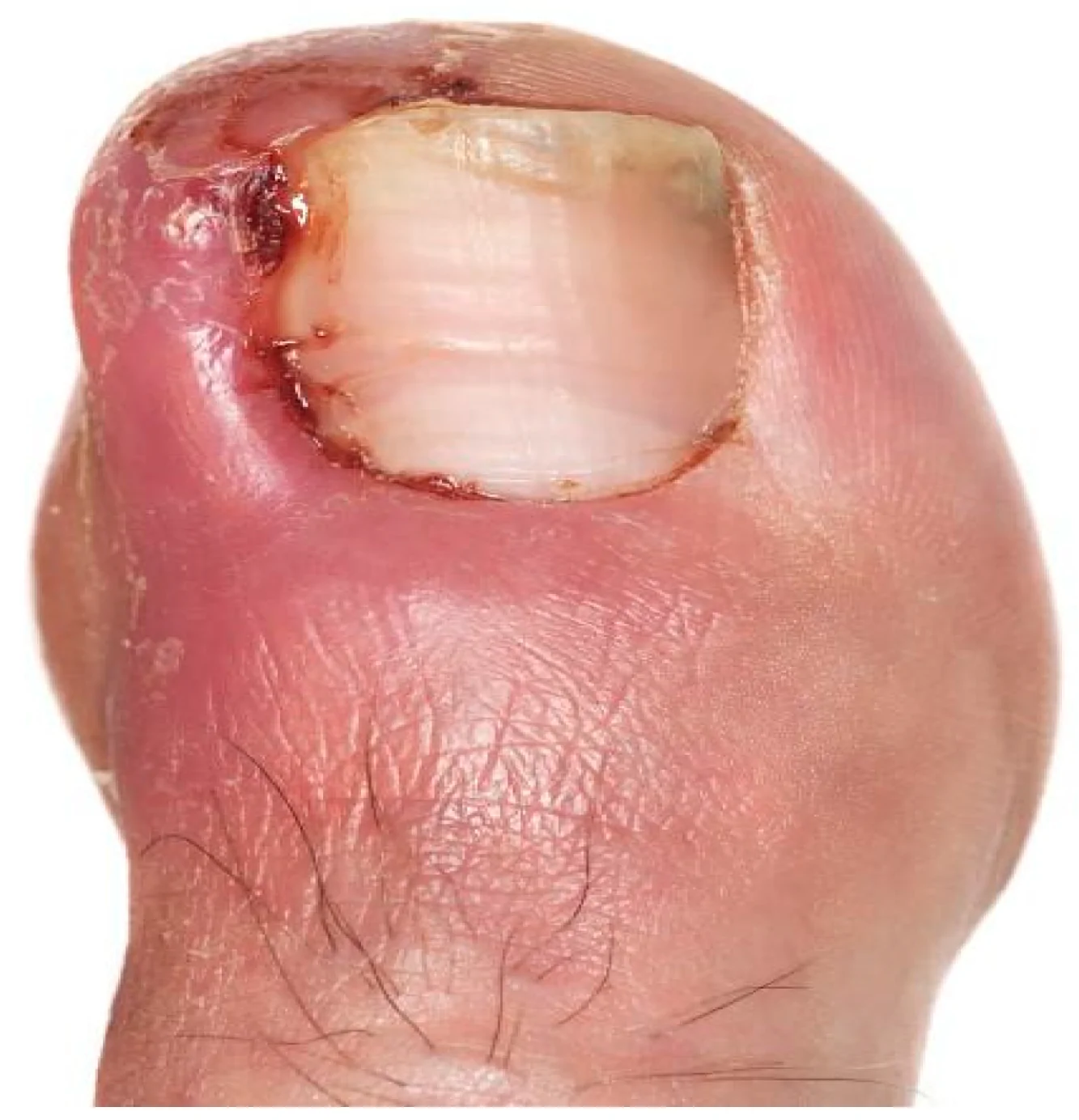
Follow-up appearance after minimal matricectomy with lateral longitudinal excision, demonstrating preservation of the remaining nail plate.

Representative postoperative appearance following minimal matricectomy is shown in Figure 3.

**Figure 4:**
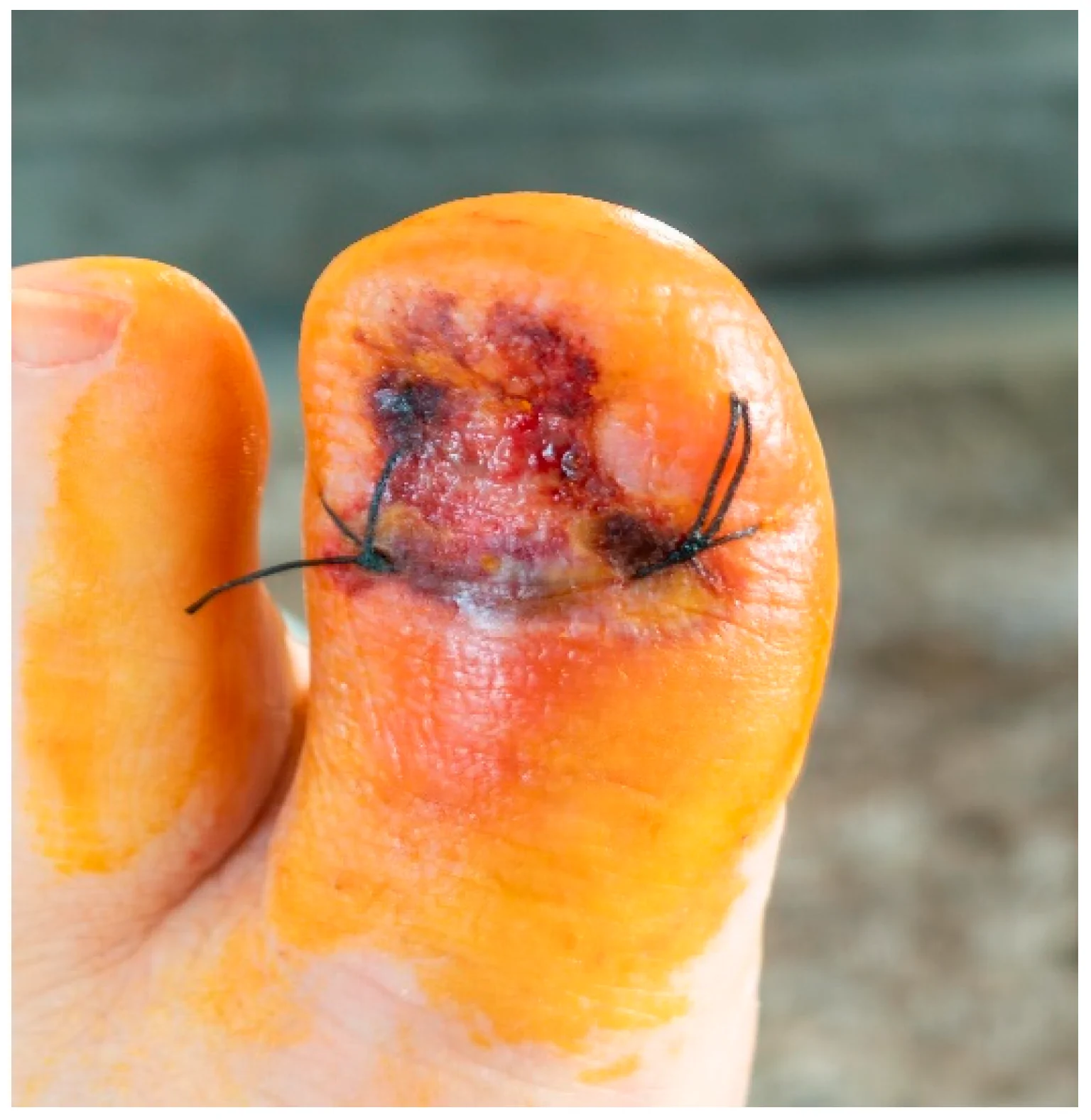
Postoperative appearance after total nail avulsion, demonstrating complete removal of the nail plate and exposure of the underlying nail bed.

The representative postoperative appearance after total nail avulsion is shown in Figure 4.

**Figure 5:**
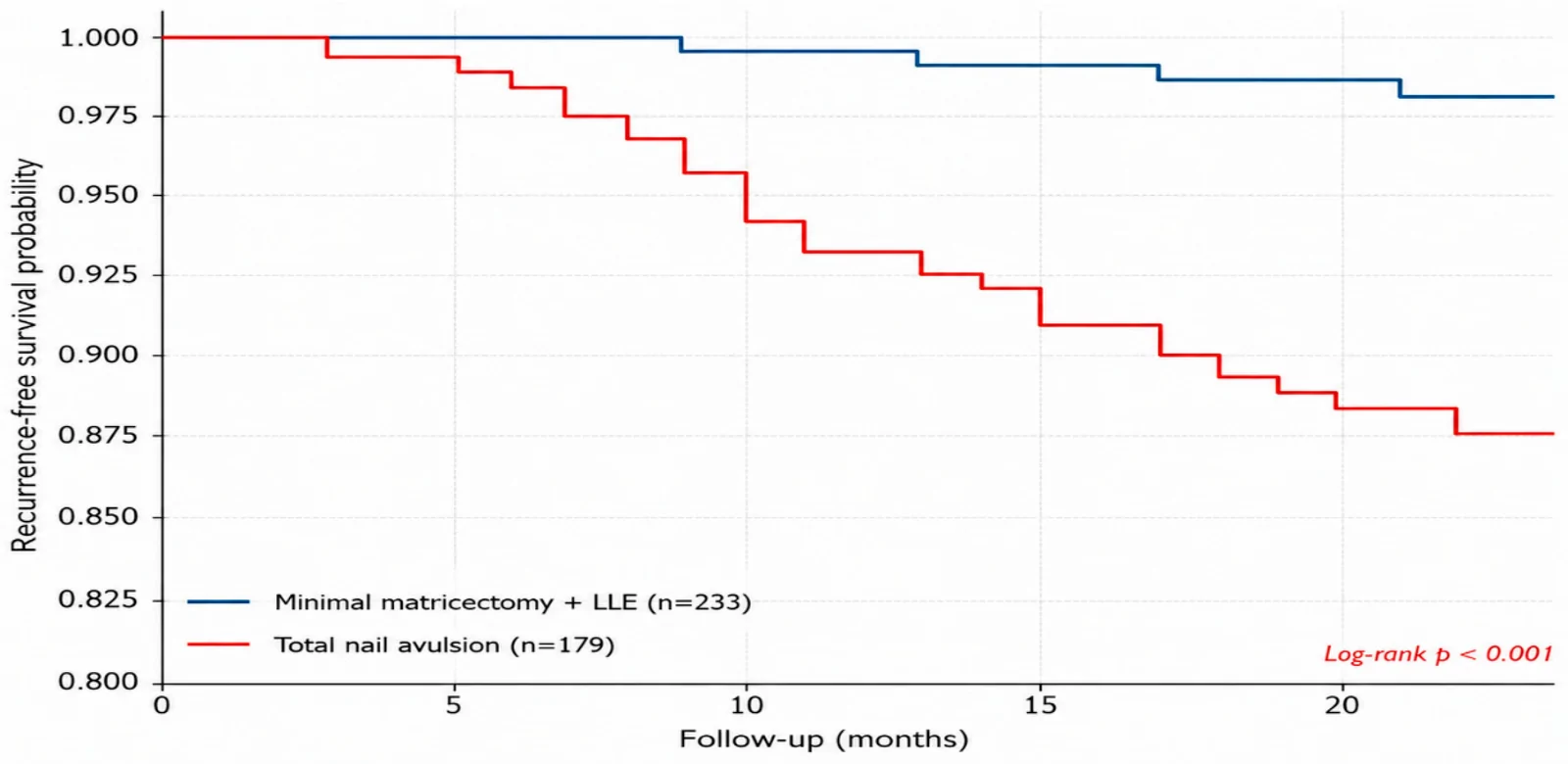
Kaplan–Meier recurrence-free survival curves comparing minimal matricectomy with lateral longitudinal excision (*n* = 233) versus total nail avulsion (*n* = 179). Groups were compared using the log-rank test.

Kaplan–Meier analysis showed significantly higher recurrence-free survival in the minimal matricectomy group throughout follow-up (log-rank *p* < 0.001; Figure 5).

## 4. Discussion

The present analysis demonstrates that a nail-preserving surgical strategy—minimal matricectomy combined with lateral longitudinal excision—is associated with a substantially lower recurrence rate when compared with total nail avulsion, and that this association persisted after propensity score adjustment for measured baseline confounders. In addition to this primary outcome, the technique showed favorable associations across multiple secondary parameters, including postoperative pain intensity, duration of healing, infection frequency, reintervention need, time to return to activity, and overall patient satisfaction. Taken together, these findings indicate that a more targeted, structure-preserving approach may yield broader clinical benefits beyond recurrence control alone, and that this benefit is unlikely to be fully explained by baseline imbalance between groups.

Recurrence remains an important consideration in the management of ingrown toenail. Total nail avulsion without matrix treatment removes the nail plate but does not directly modify the corresponding matrix or permanently alter the nail fold–nail plate interface, which may contribute to subsequent recurrence [6,7,8]. In the present study, total nail avulsion was included as a comparator because it had been performed in a substantial proportion of patients within the participating centers during the study period, rather than because it was considered the contemporary standard of care. Reported recurrence rates after total nail avulsion vary considerably but are consistently non-negligible across different clinical settings [3,7,8]. Within this context, the markedly lower recurrence observed in the present cohort, which remained statistically significant after propensity score adjustment for measured baseline covariates, supports the concept that addressing the causative mechanical factors may be more effective than complete nail removal alone [5,9,10].

Chemical matricectomy with phenol remains one of the most extensively studied matrix-ablative techniques, and several recent studies help contextualize the present findings. In a large retrospective series spanning more than a decade, Acar et al. found no significant differences in recurrence, complication rates, or patient satisfaction among surgical, sodium hydroxide, and phenol-based matricectomy [14]. A systematic review and network meta-analysis by Chang and Lin, comparing phenol, sodium hydroxide, and trichloroacetic acid, similarly reported that partial nail avulsion combined with phenolization achieved lower recurrence than avulsion alone, without a clear superiority of any single chemical agent [15]. A recent prospective comparative study likewise demonstrated markedly lower recurrence with phenol-assisted partial avulsion than with avulsion alone [16], while a triple-arm trial comparing phenol, trichloroacetic acid, and radiofrequency ablation found broadly comparable safety and satisfaction profiles across the three modalities [17]. Recent modifications aimed at improving the cosmetic outcome of partial matricectomy suggest that even chemically ablative techniques continue to be refined to reduce their traditional aesthetic drawbacks [18]. Randomized clinical trials by Muriel-Sánchez et al. have further examined phenol-based matricectomy, comparing it with an aesthetic reconstruction technique and evaluating the effect of phenol application time on recurrence and healing outcomes [19,20]. Collectively, these studies support the use of matrix-directed procedures for reducing recurrence compared with nail avulsion alone, although direct comparisons among individual matrix-directed techniques remain limited.

From a mechanistic perspective, the described surgical approach focuses on altering the anatomical interface between the nail plate and the lateral nail fold. By combining limited matrix intervention with controlled soft tissue modification, the procedure appears to reduce localized pressure and repetitive irritation. This structural adjustment may interrupt the cycle of inflammation and tissue overgrowth that contributes to recurrence. Similar principles are well established in nail-preserving and matrix-directed surgical strategies, where the goal is not only removal of pathological tissue but also correction of the underlying biomechanical imbalance [5,10,11]. Nevertheless, due to the observational design of the current study, these interpretations should be considered as supportive rather than definitive explanations.

An important aspect of the present study is the inclusion of multiple outcome measures beyond recurrence alone. The observed reductions in postoperative pain and healing time, along with lower reintervention need, faster return to activity, and improved patient satisfaction, highlight the potential advantages of less invasive techniques in terms of recovery and patient experience. These findings are consistent with previous reports suggesting that preservation of nail and surrounding structures may contribute to improved postoperative comfort and reduced morbidity [10,11,12,13]. In contrast, complete nail removal exposes the nail bed and may prolong tissue repair, which can negatively affect both recovery time and cosmetic results.

An important methodological consideration of this study is that a fully parameterized multivariable outcome regression was not undertaken, given the relatively low number of recurrence events, which would have limited the stability and reliability of such a model. To mitigate this limitation, a propensity score-based sensitivity analysis (IPTW) was performed as described in Section 2.3; the treatment effect estimate remained consistent in direction and magnitude after weighting, which reduces (without eliminating) concern that the observed association between surgical technique and recurrence reflects confounding by the measured baseline variables. Baseline disease severity, assessed using the Heifetz staging system, showed no statistically significant between-group difference before weighting and achieved adequate balance after IPTW, which further reduces the likelihood that severity-related selection bias fully accounts for the observed associations. Residual confounding by unmeasured variables cannot be excluded, and the crude comparison—rather than the adjusted one—was retained as the primary analysis given the limited event count.

Several limitations should be acknowledged. The non-randomized allocation of treatment introduces a risk of selection bias, as surgical decisions were influenced by clinical judgment and surgeon preference; while propensity score weighting addresses imbalance in measured covariates, it cannot account for unmeasured confounders. Although no statistically significant differences were observed in the reported baseline characteristics and Heifetz stage distribution, the possibility of residual confounding cannot be fully excluded. Additionally, the retrospective study design and reliance on existing clinical records limit causal inference and may introduce information bias. The study also did not include certain outcome measures, such as long-term cosmetic assessment or quality-of-life evaluation, which could provide further insight into treatment effectiveness. Finally, although the follow-up period was adequate for detecting early and intermediate recurrences, longer observation may be required to fully evaluate late recurrence patterns, and the Kaplan–Meier estimates beyond the median follow-up duration should be interpreted with caution given the smaller number of patients remaining at risk at later time points.

Despite these limitations, the study provides valuable data derived from a relatively large patient population across two clinical centers, reflecting routine practice conditions, with an analytic approach designed to address—rather than ignore—the non-randomized nature of treatment allocation. The results contribute to the evolving perspective that surgical management of ingrown toenail should focus not only on removal of affected tissue but also on correction of the underlying structural and mechanical factors.

Future investigations should prioritize prospective randomized designs with standardized outcome definitions, and where randomization is not feasible, should incorporate propensity score or target trial emulation approaches with a richer covariate set (including unmeasured factors such as detailed footwear and nail-care behavior), to allow more definitive comparisons between techniques. Such studies would help clarify the optimal surgical approach and support the development of evidence-based treatment algorithms.

## 5. Conclusions

Minimal matricectomy combined with lateral longitudinal excision was associated with lower recurrence, faster healing, fewer reinterventions, and greater patient satisfaction than total nail avulsion. The association with recurrence remained statistically significant after propensity score adjustment for measured baseline covariates. Prospective randomized trials comparing this technique with contemporary matrix-directed procedures are warranted.

## Figures and Tables

**Table 1:** Baseline demographic and clinical characteristics of patients.

Variable	Minimal Matricectomy Group (*n* = 233)	Nail Avulsion Group (*n* = 179)	*p*-Value
Age (mean ± SD)	29.1 ± 10.2	30.4 ± 9.8	0.32
Male (%)	58.5%	56.9%	0.74
Female (%)	41.5%	43.1%	—
Diabetes mellitus (%)	6.2%	6.7%	0.88
Hypertension (%)	8.1%	7.5%	0.81
Respiratory disease (%)	3.4%	3.9%	0.77
Obesity (%)	2.4%	2.8%	0.79
Improper footwear habit (%)	18.5%	21.2%	0.52
Improper nail trimming (%)	14.2%	16.8%	0.48
Prior nail intervention (%)	9.0%	10.6%	0.61

Values are presented as mean ± standard deviation or percentage (%), as appropriate. Comparisons between groups were performed using the chi-square test for categorical variables and Student’s *t*-test for continuous variables. A *p*-value < 0.05 was considered statistically significant. No statistically significant differences were observed between groups for the reported baseline variables.

**Table 2:** Heifetz stage distribution by treatment group.

Heifetz Stage	Minimal Matricectomy (*n* = 233)	Nail Avulsion (*n* = 179)	*p*-Value
Stage I	35.6% (*n* = 83)	33.5% (*n* = 60)	
Stage II	47.6% (*n* = 111)	49.7% (*n* = 89)	
Stage III	16.7% (*n* = 39)	16.8% (*n* = 30)	
Overall	100%	100%	0.87

Disease severity was retrospectively classified according to the Heifetz staging system based on documentation in patient records. Comparison performed using the chi-square test.

**Table 3:** Comparison of recurrence rates and clinical outcomes.

Outcome	Minimal Matricectomy (*n* = 233)	Nail Avulsion (*n* = 179)	*p*-Value	OR (95% CI)
Recurrence (%)	1.7% (*n* = 4)	12.3% (*n* = 22)	<0.001	8.0 (2.7–23.7)

Recurrence was defined as recurrent penetration of the nail plate into the lateral nail fold associated with pain, inflammation, granulation tissue, or the requirement for repeat intervention. Odds ratio (OR) with 95% confidence interval (CI) was calculated to estimate the association between treatment group and recurrence. Statistical comparisons were performed using the chi-square test. A *p*-value < 0.05 was considered statistically significant. This crude comparison is the primary analysis; see Table 4 for the propensity score-weighted sensitivity analysis.

**Table 4:** Propensity score-weighted (IPTW) sensitivity analysis.

Covariate	SMD—Before Weighting	SMD—After IPTW
Age	0.13	0.03
Sex	0.03	0.01
Diabetes mellitus	0.02	0.01
Obesity/BMI	0.05	0.02
Heifetz stage	0.06	0.02
Footwear habit	0.11	0.04
Improper nail trimming	0.09	0.03
Treating center	0.08	0.02
Prior nail intervention	0.07	0.02

**IPTW-adjusted outcome:** Recurrence—total nail avulsion vs. minimal matricectomy, adjusted OR 7.4 (95% CI 2.6–21.3), *p* < 0.001. Standardized mean differences (SMD) < 0.10 indicate adequate covariate balance after inverse probability of treatment weighting (IPTW). Propensity scores estimated via multivariable logistic regression on the listed covariates.

**Table 5:** Clinical outcomes comparison between groups.

Outcome	Minimal Matricectomy	Nail Avulsion	*p*-Value
Postoperative pain (VAS, day 3)	2.1 ± 0.9	4.3 ± 1.2	<0.001
Healing time (days)	14.7 ± 4.2	23.8 ± 7.7	<0.001
Infection rate (%)	3.2%	9.8%	0.01
Patient satisfaction (1–5)	4.6 ± 0.5	3.2 ± 0.8	<0.001
Reintervention rate (%)	1.3% (*n* = 3)	9.5% (*n* = 17)	<0.01
Return to normal activity (days, median)	5	9	<0.001

VAS: Visual Analog Scale. Healing time defined as complete epithelialization. Infection defined as need for antibiotic therapy. Patient satisfaction assessed using a 5-point Likert scale (1 = very dissatisfied, 5 = very satisfied). Reintervention defined as any repeat surgical or chemical intervention for the same lesion. Return to normal activity defined as resumption of unrestricted weight-bearing and usual daily/occupational activity.

## Data Availability

The data presented in this study are available on reasonable request from the corresponding author, subject to institutional and ethics committee restrictions related to patient confidentiality.
